# Underwater Target Detection Based on Parallel High-Resolution Networks

**DOI:** 10.3390/s23177337

**Published:** 2023-08-23

**Authors:** Zhengwei Bao, Ying Guo, Jiyu Wang, Linlin Zhu, Jun Huang, Shu Yan

**Affiliations:** 1 College of Automation, Nanjing University of Information Science & Technology, Nanjing 210044, China; 2 Jiangsu Collaborative Innovation Center of Atmospheric Environment and Equipment Technology (CICAEET), Nanjing 210044, China

**Keywords:** underwater, target detection, parallel high-resolution networks, attention mechanism, receptive field augmentation

## Abstract

A parallel high-resolution underwater target detection network is proposed to address the problems of complex underwater scenes and limited target feature extraction capability. First, a high-resolution network (HRNet), a lighter high-resolution human posture estimation network, is used to improve the target feature representation and effectively reduce the semantic information lost in the image during sampling. Then, the attention module (A-CBAM) is improved to capture complex feature distributions by modeling the two-dimensional space in the activation function stage through the introduction of the flexible rectified linear units (FReLU) activation function to achieve pixel-level spatial information modeling capability. Feature enhancement in the spatial and channel dimensions is performed to improve understanding of fuzzy targets and small target objects and to better capture irregular and detailed object layouts. Finally, a receptive field augmentation module (RFAM) is constructed to obtain sufficient semantic information and rich detail information to further enhance the robustness and discrimination of features and improve the detection capability of the model for multi-scale underwater targets. Experimental results show that the method achieves 81.17%, 77.02%, and 82.9% mean average precision (mAP) on three publicly available datasets, specifically underwater robot professional contest (URPC2020, URPC2018) and pattern analysis, statistical modeling, and computational learning visual object classes (PASCAL VOC2007), respectively, demonstrating the effectiveness of the proposed network.

## 1. Introduction

With the development of computer vision and the exploitation of marine resources, biological detection in underwater environments has become a hot research topic with applications in underwater robotics [1], underwater exploration [2], and marine research [3]. However, compared to terrestrial scenes [4], underwater images suffer from problems such as color bias [5], low contrast [6], blur [7], and noise [8], resulting in the loss of clear contour and texture information in images, which limits the accuracy of target detection. Therefore, accurate detection of marine organisms in complex underwater environments remains a challenge. To this end, a parallel high-resolution underwater target detection network is proposed to improve the accuracy and efficiency of target detection given the complexity of underwater scenes, limited target feature extraction capability, fuzzy targets, and small targets.

Currently existing target detection models can be classified into two types: anchor-free and anchor-based. Anchor-based detectors include the region-based convolutional network (R-CNN) series [9,10,11,12], the You Only Look Once (YOLO) series [13,14,15], and the Single Shot MultiBox detector (SSD) series [16,17], which provide a priori knowledge of the model by using predefined anchor boxes and have achieved better performance. However, the anchor box introduces redundant negative samples, leading to a reduction in inference speed. Anchor-free detectors such as CenterNet [18], CornerNet [19], and fully convolutional one-stage (FCOS) [20] and end-to-end object detection with transformers (DETR) [21] discard the anchor box and treat the target as a key point for prediction. With effective training, the anchor-free detector can compete with the anchor-based detector in terms of performance and has a better generalization capability.

Recent research on underwater objects, for example, Victor et al. [22] provided an overview of various applications of federated learning in the internet of underwater things (IoUT), as well as its challenges and unsolved issues, have indicated future research prospects. The IoT framework emphasizes the significance of data security and privacy. Meng et al. [23] argued that in order to discover the valuable hidden data detected by an automatic underwater vehicle (AUV) swarm, it is necessary to aggregate the data detected by the AUV swarm to generate a powerful machine learning model and introduced federated learning into the collaboration of an AUV swarm. To further reduce the constraints of scarce underwater communication resources on federated learning and to mitigate the lag effect, an asynchronous federated learning method was developed. Bhattacharya et al. [24] first discussed blockchain technology and the Internet of Things (IoT), pointing out the benefits of integrating blockchain technology with IoT systems and providing an overview of various applications of blockchain-enabled IoUT systems, their respective challenges, and possible future directions. Finally, this work identifies key aspects of IoUT systems and enables researchers to address challenges using blockchain technology to improve the accuracy and efficiency of underwater target detection and to promote the development of underwater target detection by integrating research results and improvement strategies from different fields.

In the field of underwater environments, researchers have made important contributions by proposing various target detection algorithms [25,26,27]. For example, Zhou et al. [28] combined image enhancement techniques with an extended visual geometry group (VGG16) feature extraction network in 2017, using a faster region-based convolutional neural network (Faster R-CNN) and feature mapping to detect and identify underwater biological targets in a URPC dataset. Villon et al. [29] combined a histogram of oriented gradient (HOG) and a support vector machine (SVM) to detect coral reef fish in captured images, solving the problem of underwater accuracy degradation caused by target occlusion and overlap in the images. Chen et al. [30] introduced a novel sample distribution-based weighted loss function called the Invert Multi-Class AdaBoost (IMA) in 2020 to mitigate the adverse effects of noise on detection performance. Ma et al. [31,32] developed lightweight models by pruning existing target detection models (e.g., SDD) or integrating lightweight backbones (e.g., MobileNet). Li et al. [33] added an extra detection head to the YOLOv5 model to improve multi-scale detection and small target detection accuracy. Zhang et al. [34] combined an attention mechanism to learn target features and increase the receptive field to improve the detection accuracy and robustness of underwater small target detection. Yao et al. [35,36] used residual networks as a backbone network to improve the efficiency of sea cucumber feature extraction. Jiang et al. [37] also proposed a method called the Channel Sharpening Attention Module (CSAM) for further fusion of advanced image information. A summary of related work methods is shown in Table 1.

The task of target detection becomes particularly difficult in the underwater environment due to blurred feature information, severe color distortion, and low visibility, requiring simultaneous localization and classification. In order to improve the accuracy and portability of underwater target detection capabilities, we have chosen to improve the classical CenterNet model in the anchor-free family and propose a parallel high-resolution network model to address the challenges faced in this domain. The effectiveness of our proposed model is demonstrated by conducting experiments on underwater images.

The innovations in this study are as follows: (1) aiming at the problem of complex underwater scenes and limited target feature extraction capability, a lighter high-resolution human posture estimation network (HRNet) [38] is used to enhance the target feature representation and effectively reduce the semantic information lost in the image sampling process; (2) for the problem of fuzzy and small targets, by introducing the FReLU [39] activation function, the improved convolutional block attention module (CBAM) [40] structure captures the complex feature distribution by modeling the two-dimensional space in the activation function stage to achieve the ability to model spatial information at the pixel level while performing feature enhancement in the spatial dimension and channel dimension; (3) we constructed a receptive field augmentation module (RFAB) to further obtain sufficient semantic information and rich detail information to further enhance the robustness of the features and discriminative ability of the model for underwater multi-scale target detection.

## 2. Related Work

From the literature, it can be seen that by having a multi-resolution network parallel connection structure and fusing feature maps of different resolutions so that each resolution feature map can accept information from other resolution feature maps many times, output feature maps can be obtained that contain both high-resolution positional information and low-resolution semantic information, which can enhance the expression of target features and reduce the semantic information lost in the sampling process. However, the separate use of low- and high-resolution features still cannot meet our needs for high-quality recognition. If the attention mechanism can be properly used to enhance the target information in the feature map from both spatial and channel dimensions, it can guide the model to increase its attention to key information. It also improves its activation function to model the 2D space in the activation function stage to capture the complex feature distribution. In addition, the receptive field module demonstrates the model’s ability to obtain rich semantic information about the target context.

### 2.1. High-Resolution Network

In convolutional neural networks, high-resolution images contain richer detail information and play a crucial role in target detection. However, high-resolution feature maps also have a higher computational cost, resulting in more operations. In addition, in the deeper layers of the model, low-resolution feature maps contain rich semantic information that helps improve the model’s ability to detect targets.

HRNet fuses low-resolution feature maps with high-resolution feature maps by connecting networks of different resolutions in parallel, so that the feature extraction network outputs high-resolution feature maps that contain rich semantic information. The feature maps of different resolutions are obtained through different branches of the image, and the feature maps of each branch are fused with the feature maps of all scales in the previous stage, so that the feature maps of each branch take into account both high- and low-resolution feature information, thus retaining more target feature information.

The key feature of HRNet is that it maintains high resolution throughout the model’s processing of the image and generates multiple low-resolution feature maps simultaneously, which are used to augment the feature information of the high-resolution feature maps. Unlike previous networks, HRNet does not use extended convolution or upsampling to pass low-resolution features to recover the high-resolution representation. Instead, HRNet maintains the high-resolution representation throughout the modeling process and continuously performs cross-scale fusion to learn richer feature representations. HRNet is designed using a stage-by-stage incremental strategy, starting with a high-resolution subnet and then gradually adding new subnets in parallel to form multiple stages, with the resolution of the current subnet being 1/2 the resolution of the previous subnet. This process is repeated multiple times, eventually forming four stages. Based on these considerations, HRNet is a lighter weight network for high-resolution human posture estimation.

### 2.2. Attention Mechanism

Attention mechanisms are becoming popular in various fields and are often applied in a weighted manner. In computer vision tasks, where different regions of an image or video frame may have different levels of importance, attention mechanisms have been found to allow more flexible processing of visual information. The basis of this mechanism is similar to the human selective attention mechanism. Humans tend to ignore irrelevant information when observing objects and focus on information that is useful for decision making. Attention mechanisms are based on the idea of acquiring task-critical information. Therefore, an attention mechanism is often used to automatically select features or regions of interest during target detection.

Models that process target images contain a large number of “meaningless” regions that do not contain information about the target, which interferes with feature extraction of valid targets. To improve feature extraction, researchers have proposed attention mechanisms to guide models to focus on target regions. The design of attention mechanisms is inspired by the unique brain signal processing mechanism of human vision. When the human eye observes an image, it generates a focus of attention that concentrates on certain key regions to eliminate the interference of irrelevant information, thus improving the accuracy and efficiency of information processing.

Attention mechanisms in neural networks are plug-and-play learnable modules that work by assigning weight coefficients to values in the feature map tensor to enhance information in the focus region. They mainly include the channel attention mechanism and the spatial attention mechanism. The channel attention mechanism achieves dynamic enhancement of the features in each channel by assigning weights to the channel dimension of the feature map, while the spatial attention mechanism assigns weights in the spatial dimension and learns the dependencies of different locations on the image to dynamically enhance the features in the spatial dimension. Self-attention mechanisms, on the other hand, are a branch of attention mechanisms developed from the field of natural language processing and are unique in that they do not rely on the guidance of external information but rely on their own input to establish global dependencies to generate weight coefficients. By combining the structure of these two mechanisms, the target information in the feature map can be strengthened in terms of spatial and channel dimensions. In addition, the activation function can be enhanced to capture complex feature distributions by modeling the 2D space using the activation function stage. The FReLU activation function, which is specifically designed for vision tasks, is chosen to replace the ReLU activation function in the CBAM module. The FReLU activation extends ReLU to a two-dimensional activation function by adding a negligible spatial conditioning overhead, which improves target detection accuracy by capturing complex visual layouts in two-dimensional space. In addition, spatial conditioning implements pixel-level modeling capabilities in a simple way to capture complex visual layouts through regular convolution. FReLU, on the other hand, is a spatial context-dependent 2D condition that helps to extract the spatial layout of target features.

### 2.3. Receptive Field Module

Small targets occupy fewer pixels in images, resulting in insufficient information about their characteristics. In order to optimize the recognition effect, an optimization idea can be adopted, which is to use the information of the target’s environment to support the recognition, called the target contextual semantic information. Usually, the objects in the image do not exist independently, and there is some relationship between them and the environment. Therefore, by making full use of the target contextual semantic information, the feature representation of the target can be effectively enriched. One of the commonly used optimization methods is to expand the receptive field of the neural network by using null convolution. 

The receptive field module takes advantage of the population receptive field properties of human vision and constructs a multi-branch structure using hollow convolutional kernels of different sizes and eccentricities. This receptive field module on the input image is usually first processed with three ordinary convolution kernels of different sizes (1 × 1, 3 × 3, 5 × 5). Then, three feature maps with different receptive fields are separately obtained using hollow convolution with different centrifugal rates. By fusing these feature maps, feature enhancement results can be obtained. To reduce the computational effort, alternative convolution kernels can be used, e.g., the 5 × 5 convolution layer can be replaced by a 3 × 3 convolution layer, and the 3 × 3 convolution layer can be replaced by 1 × 3 and 3 × 1 convolution layers.

Compared to the traditional convolutional module, the feature maps produced by the lower centration branches of the module are able to condense feature information that includes parts or the target as a whole. The higher centrifugal branches can condense the feature information including the target itself and the surrounding environment. The advantage of fusing multiple branches is that the higher centrifugal branch can provide contextual semantic information to the lower centrifugal branch, while the lower centrifugal branch can compensate for the loss of detail caused by the convolutional kernel diffusion of the higher centrifugal branch.

The structure of the receptive field is borrowed and both scale and centrifugal rate are taken into account. Compared to the use of a computationally intensive and deeply layered backbone network, the module uses a lightweight backbone network. By combining the receptive field module, the receptive field augmentation module proposed in this paper achieves a stronger feature representation capability, which enables the detector to have a faster inference speed while maintaining excellent performance. In addition, the training process does not require too many complex tricks, so the training resources of the model are less.

In the existing one-stage and two-stage detectors, they usually face the dilemma of good performance but slow speed or fast speed but unsatisfactory performance. To address this problem, this paper proposes a scheme that uses a lightweight backbone and enhanced receptive field module to achieve faster inference while maintaining performance. The design elegantly combines feature fusion with different centrifugal velocities, bringing significant performance gains to the target detection task.

## 3. Methods

### 3.1. Improved Network Structure

**Figure 1:**
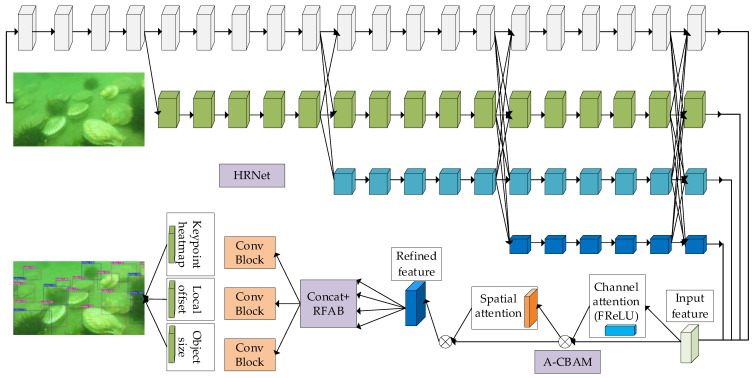
Overall network structure.

In order to effectively integrate high-resolution shallow feature information and low-resolution deep feature information, this paper proposes a network structure based on the parallel connection of multi-resolution subnetworks designed by CenterNet. The underwater object detection capability is enhanced by using a backbone network (HRNet), improving the attention module (A-CBAM), and constructing a receptive field augmentation module (RFAB). It consists of two parts: the AR-HRNet backbone network and the recognition module. The improved network structure is shown in Figure 1.

The input image is first passed through four parallel subnetworks of the HRNet, allowing for the rapid extraction of features at four different resolutions. The four different resolution features are then fed into the attention module (A-CBAM), which improves model performance by increasing the spatial and channel dimensions. In the CBAM structure, complex feature distributions are captured in the activation function stage by modeling the two-dimensional space through the introduction of the FReLU activation function to achieve the ability to model spatial information at the pixel level. It then passes through the receptive field augmentation module (RFAB) to further capture rich semantic and positional information to enhance target detection at multiple scales underwater. Finally, the fused features are passed to three separate branches to generate key point heatmaps, position offsets and target width and height, and the final detection results are obtained through decoding operations.

#### 3.1.1. Parallel High-Resolution Network

The benchmark network CenterNet adopts Hourglass-104 as the backbone network; although it can achieve high detection accuracy, the network has a complex structure and a larger number of parameters, which greatly increases the computational complexity of the network and reduces the detection speed of the network. Therefore, in order to improve the detection speed of the algorithm while maintaining high detection accuracy, this paper changes the backbone network to a HRNet network from the perspective of lightweight network structure.

Originally used for human posture estimation, HRNet is a feature extraction network designed to overcome the information loss and ambiguity problems that occur with traditional deep learning networks when processing high-resolution images. HRNet effectively improves the network’s ability to perceive detailed information by parallelizing multiple branches to process feature maps at different resolutions and by interacting and fusing between them.

**Figure 2:**
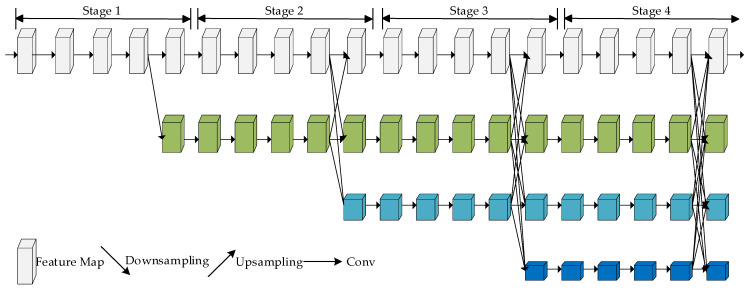
HRNet structure.

The core idea of HRNet is to maintain the transfer and use of high-resolution features. It enables the network to maintain high-resolution features at different resolutions by keeping information fluid between branches. Specifically, HRNet allows the network to maintain both global receptive field and detail information by performing resolution-by-resolution upsampling and feature fusion multiple times. This multi-level feature fusion design allows HRNet to better capture targets at different scales in target detection tasks and provide more accurate and detailed detection results. The structure of the HRNet network is shown in Figure 2.

**Figure 3:**
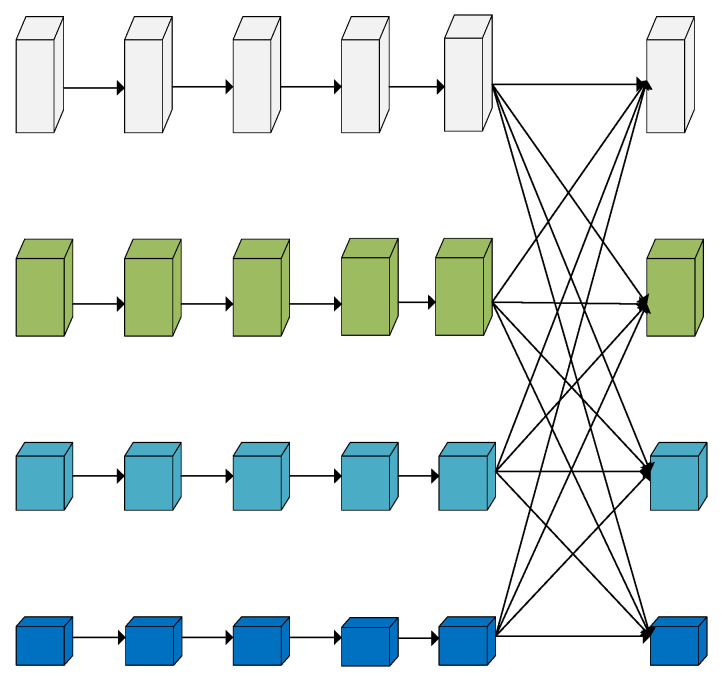
Multi-resolution module.

**Figure 4:**
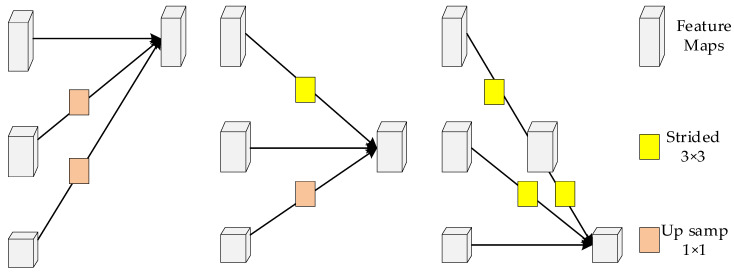
Specific fusion method.

HRNet is divided into four stages: each stage is a parallel connection of multi-resolution subnetworks and consists of a series of standard convolutions; the feature map resolution of the same subnetwork does not change with the depth of the network, while the feature map resolution of the parallel subnetworks decreases in turn by 1/2, while the number of channels increases by a factor of 2. Starting from the backbone network, the image is processed by a 3 × 3 convolution with a step size of 2. The feature map size obtained is 1/4 of the original image and the number of channels is increased from 3 to 64. A high-resolution subnetwork is constructed and the first stage consists of a subnetwork using four bottleneck [41] modules to extract features and adjusting the channels to 32. The multi-resolution module is shown in Figure 3. The second, third, and fourth stages then consist of multi-resolution modules containing 1, 4, and 3 multi-resolution modules, respectively, where the multi-resolution module consists of 4 BasicBlocks at each resolution, followed by a cross-resolution fusion module. The specific fusion method is shown in Figure 4. For the upsampling operation, the number of channels is first aligned using a 1 × 1 convolutional layer and the resolution is expanded using a neighborhood interpolation method. For the downsampling operation, a 3 × 3 convolution with a step size of 2 is used for 2-fold downsampling and two 3 × 3 convolutions with a step size of 2 are used for 4-fold downsampling.

The four subnetworks of HRNet downsample the images by 4, 8, 16, and 32 times to extract feature maps of different resolutions. The original HRNet only uses the output of the high-resolution subnetwork, but to make full use of the multi-scale feature information, this paper uses the feature maps generated by the four parallel subnetworks as the output of the backbone network. This can effectively fuse features at different scales to improve the performance and accuracy of target detection.

#### 3.1.2. Improved Attention Mechanism

**Figure 5:**
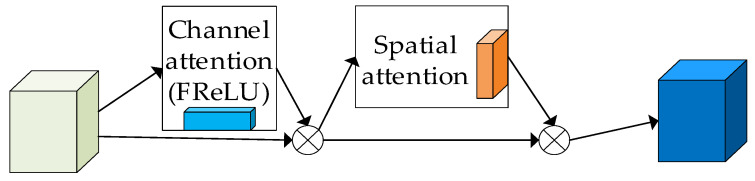
A-CBAM module.

The CBAM consists of a linear stack of the channel attention module (CAM) and the spatial attention module (SAM). This paper improves the ReLU activation function in the CBAM module by replacing it with the FReLU activation function, which is specifically designed for visual tasks. FReLU activation extends ReLU to a two-dimensional activation function by adding negligible spatial conditioning overhead, which captures complex visual layouts in two-dimensional space, improving target detection accuracy. In addition, spatial conditioning implements pixel-level modeling capability in a simple way to capture complex visual layouts through regular convolution. FReLU, on the other hand, is a spatial context-dependent 2D condition that helps to extract the spatial layout of target features. Based on this, we have designed a new attention mechanism called the A-CBAM attention mechanism. By applying the A-CBAM attention mechanism, we expect to further improve the target detection performance. Its structure is shown in Figure 5.

**Figure 6:**
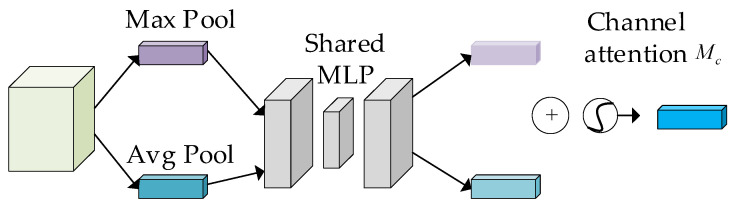
Channel attention module.

The function of the aforementioned channel attention module is to compress the spatial dimensionality of the feature map by applying average pooling and max pooling operations based on width and height, transforming it into a one-dimensional vector and then processing it. Average pooling and max pooling can be used to aggregate the spatial information of the feature map. The feature vectors are then passed to the fully connected layer, where the spatial dimensions of the input feature map are compressed and then summed and merged element by element. Finally, the channel attention map is generated by applying a sigmoid activation function. The structure is shown in Figure 6.

Its formula is:

(1) $$M_{c}(F)=\sigma (MLP(AvgPool(F))+MLP(MaxPool(F)))$$

where $M_{c}(F)$ is the channel attention feature, σ is the sigmoid activation function, MLP is a 2-layer neural network, *F* is the input, *AvgPool* is the average pooling, and *MaxPool* is the maximum pooling. 

**Figure 7:**
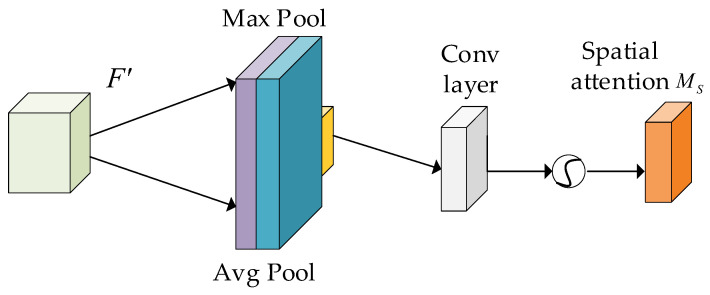
Spatial attention module.

To obtain the attentional features of the feature map in the spatial dimension, we performed global maximum pooling and global average pooling on the feature map output using the channel attention module to rescale the dimension of the feature map from H × W to 1 × 1. Next, we performed convolution using a 7 × 7 convolution kernel and applied the sigmoid activation function to reduce the dimensionality of the feature map. We then merged the feature map normalized by the sigmoid activation function with the feature map output by the channel attention module to complete the rescaling of the feature map in both spatial and channel dimensions. The structure is shown in Figure 7.

Its formula is:

(2) $$M_{S}(F)=\sigma \left(f^{7\times 7}([AvgPool(F);MaxPool(F)])\right)$$

where $M_{S}(F)$ is the spatial attention feature and $f^{7\times 7}$ is the convolution of the 7 × 7 convolution kernel.

A key component of CBAM is the Multilayer Perceptron (MLP), a module that uses ReLU as the activation function for implementing non-linear activation. The introduction of the ReLU function solves the gradient disappearance problem in the backpropagation algorithm. However, in order to achieve pixel-level spatial information modeling capability and improve accuracy in the activation function stage, this paper replaces the original ReLU activation function with a FReLU activation function specifically designed for vision tasks. The ReLU function is represented as y=max(x,0), while the FReLU function is of the form y=max(x,T(x)), where T(⋅) is the two-dimensional spatial representation. By using two-dimensional spatial information, FReLU can better capture complex visual layouts and improve the accuracy of target detection. Unlike the fixed zero-value condition of ReLU, FReLU is a two-dimensional condition that depends on the spatial context and helps to extract the spatial layout of the target features. Specifically, the two-dimensional condition depends on the spatial context of each pixel and uses the max(⋅) function to obtain the maximum value between input x and the condition. The FReLU activation function is defined as shown below:

(3) $$f\left(x_{c},i,j\right)=\max\left(x_{c},i,j,T\left(x_{c},i,j\right)\right)$$

In the above formulation, $T\left(x_{c},i,j\right)$ denotes the two-dimensional condition and we use a parametric pooling window to introduce spatial dependence. To achieve spatial dependence, we use a highly optimized depth-separable convolution operator and a batch normalization layer. The specific unfolding $T\left(x_{c},i,j\right)$ is computed as shown below:

(4) $$T\left(x_{c},i,j\right)=x_{c,i,j}^{w}\cdot p_{c}^{w}$$

**Figure 8:**
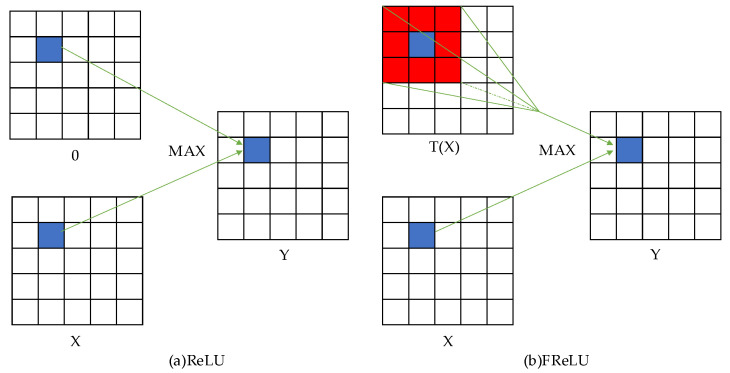
Comparison of ReLU activation function and FReLU activation function. (**a**) ReLU activation function. (**b**) FReLU activation function.

In the above equation, (i,j) denotes the pixel position in two-dimensional space, *c* denotes the *c*-th channel, and $x_{c,i,j}^{w}$ denotes the input pixel as a non-linear activation function for the *c*-th channel at the two-dimensional spatial position (i,j). A parameterized pool window is used here, where $p_{c}^{w}$ represents the coefficients shared by this window in the same channel. At the same time, the ReLU activation function was compared with the FReLU activation function, as shown in Figure 8.

The CBAM structure with the addition of the FReLU activation function is called A-CBAM. FReLU has a stronger context capture capability, resulting in a better understanding of fuzzy targets and small target objects. In complex cases, the A-CBAM structure is able to capture irregular and detailed object layouts better than the CBAM structure.

#### 3.1.3. Improved Receptive Field Augmentation Module

The receptive field is the size of the region in which the pixel points on the feature map output by each layer of the convolutional neural network are mapped onto the input image. Neurological studies have shown that in the human visual cortex, the size of the population receptive field correlates with retinal eccentricity and that the size of the population receptive field varies in different retinal regions. To simulate this finding and to improve the deep features learned by lightweight convolutional neural network models, Liu et al. [42] proposed the receptive field block (RFB). This block can improve the ability of networks to learn deep features by simulating the properties of population receptive fields in the human visual system.

**Figure 9:**
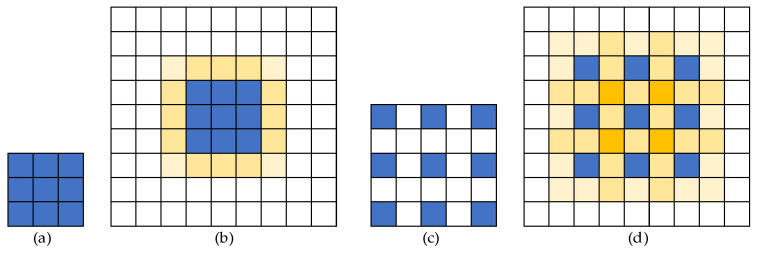
Comparison of ordinary convolution and dilated convolution. (**a**) The 3 × 3 convolution kernel. (**b**) Schematic of the response to a 3 × 3 convolution operation. (**c**) The 3 × 3 dilated convolution kernel. (**d**) Schematic of the response to a 3 × 3 dilated convolution operation.

The receptive field module combines the parallel convolution of dilated convolution [43] and InceptionNet [44] to expand the receptive field of the feature layers. Null convolution is proposed to achieve receptive field expansion without reducing the size (resolution) of the feature map, allowing the output of each convolution to contain a greater range of information. A comparison of the dilated convolution with the ordinary convolution is shown in Figure 9.

**Figure 10:**
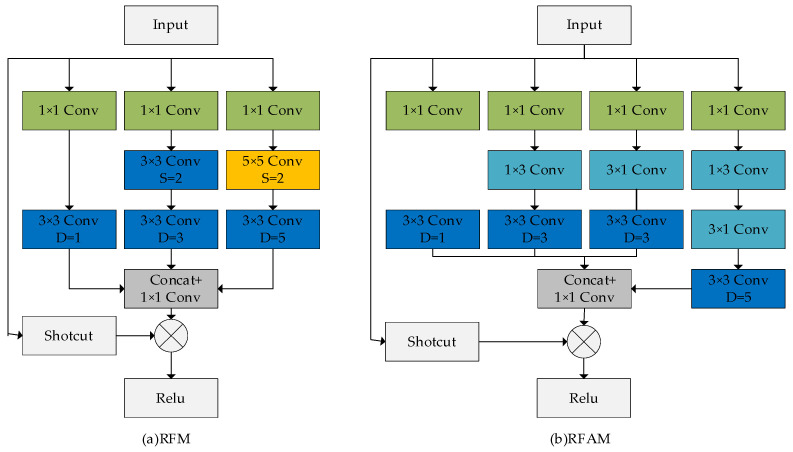
Comparison of receptive field module and receptive field augmentation module. (**a**) Receptive field module (RFM). (**b**) Receptive field augmentation module (RFAM).

The original RFB has three branches, each with a different sized convolution kernel; the sizes of the convolution kernels are 1 × 1, 3 × 3, and 5 × 5, and the end of the branch has a dilated convolution layer; the dilated rates are 1, 3, and 5, respectively, which determines the spacing of the convolution kernel processing data. The larger the dilated rate, the larger the spacing of the convolution kernel processing data. To adapt to different situations, we propose the RFAM structure, which has more branches than RFM and contains four branches, with different branches corresponding to the respective convolutional and dilated layers. RFAM uses 1 × 1 convolutional kernels in each branch to adjust the number of channels and uses a series of 3 × 3 convolutions instead of 5 × 5 convolutions to reduce the number of parameters in the model to increase the detection speed while increasing the model’s nonlinearity. In addition, we replaced the original 3 × 3 convolution with a 1 × 3 convolution and a 3 × 1 convolution. The results of the 4 branches are concatenated and passed through a 1 × 1 convolution and then fed into the activation function with a residual edge as input. The effect of the residual edge is to allow the input image signal to propagate directly from bottom to top, mitigating the gradient divergence caused by an overly deep network. Its calculation is shown below:

(5) $$X_{\mathrm{out}\;}=\tau \left(X_{\mathrm{in}\;}⊗\;\epsilon (Br1+Br2+Br3)\;\times \;\right.scale)$$

where $X_{\mathrm{in}\;}$ denotes the input features and *Br*1, *Br*2, and *Br*3 denote the outputs of the three branches. The + denotes the feature fusion operation. We use the symbol ϵ to denote the process of adjusting the number of channels by 1 × 1 convolution. scale denotes the weight of the residual edge linear operation; here, we take 0.1 as the weight value. The ⊗ operator denotes summation by element. Finally, the symbol τ denotes the ReLU activation function. A comparison of the receptive modules is shown in Figure 10.

#### 3.1.4. Loss Function

The total loss function in this paper’s algorithm can be divided into three components: heat map loss, center bias loss, and width–height loss, and the three loss functions are weighted and summed.

Heat map loss $L_{k}$ is a function with a focal loss penalty mechanism, defined as:

(6) $$L_{k}=\left\{\begin{array}{l} \frac{-1}{N}\sum_{x,y,c}{\left(1-{\hat{Y}}_{x,y,c}\right)}^{\alpha }\log\left({\hat{Y}}_{x,y,c}\right),Y_{x,y,c}=1\\ \frac{-1}{N}\sum_{x,y,c}{\left(1-Y_{x,y,c}\right)}^{\beta }{\left({\hat{Y}}_{x,y,c}\right)}^{\alpha }\log\left(1-{\hat{Y}}_{x,y,c}\right),\;\mathrm{otherwise}\; \end{array}\right.$$

In CenterNet, the hyperparameters *a* and *β* of the focal losses were considered as well as the number of key points in the image *N* and the predicted value ${\hat{Y}}_{x,y,c}$. According to the original method, we chose *α* = 2 and *β* = 4 and normalized all the focal losses. In addition, when dealing with easily classifiable samples, we reduced their training weight appropriately.

During the downsampling process of the model, the resolution of the feature maps extracted by the backbone network decreases due to the output span, which leads to the loss of details in the images and, thus, causes a bias in the prediction results. Therefore, we introduced the centroid bias value $\hat{O}\in R^{\frac{W}{R}\cdot \frac{H}{R}\cdot 2}$, used the $L_{1}$ loss function for training, and defined the centroid bias loss $L_{\mathrm{off}}$ as:

(7) $$L_{\mathrm{off}}=\frac{1}{N}\sum_{p}\left|{\hat{O}}_{\tilde{p}}-\left(\frac{p}{R}-\tilde{p}\right)\right|$$

where *R* is the sampling factor, typically *R* = 4, *p* is the target frame centroid, and $\left(\frac{p}{R}-\tilde{p}\right)$ is the bias value.

Let the target bounding box coordinates be $\left(x_{1}^{\left(k\right)},y_{1}^{\left(k\right)},x_{2}^{\left(k\right)},y_{2}^{\left(k\right)}\right)$; then the centroid can be denoted as $\left(\frac{x_{1}^{\left(k\right)}+x_{2}^{\left(k\right)}}{2},\frac{y_{1}^{\left(k\right)}+y_{2}^{\left(k\right)}}{2}\right)$, and the same $L_{1}$ loss function is used for training, with the width–height loss $L_{size}$ defined as:

(8) $$L_{\mathrm{size}\;}=\frac{1}{N}\sum_{k=1}^{N}\left|{\hat{S}}_{pk}-s_{k}\right|$$

Combining the above types of losses gives the total loss $L_{\det\;}$, which is expressed as:

(9) $$L_{\det\;}=L_{k}+\lambda_{\mathrm{size}\;}L_{\mathrm{size}}+\lambda_{\mathrm{off}\;}L_{\mathrm{off}\;}$$

where the weights $\lambda_{\mathrm{off}\;}$ and $L_{\mathrm{size}}$ are taken as 1 and 0.1, respectively.

## 4. Experiments and Analysis

In this section, we validate the effectiveness of the proposed method through several sets of experiments. First, we conducted experiments on four types of underwater image datasets, including comparison experiments with popular target detection algorithms, ablation studies, and comparison experiments with existing underwater target detection algorithms. Then, we conducted experiments on the PASCAL VOC2007 dataset to verify that our method not only performs well on underwater datasets but is also applicable to standard datasets.

### 4.1. Training Details

The experimental environment is Python 3.8, Pytorch 1.7.0, Torchvision 0.7.0, and the rest of the Python modules can be downloaded and installed from third parties. The hardware consists of an Intel^®^ Core™ i9-10900X CPU and an NVIDIA RTX308Ti graphics card. The Intel^®^ Core™ i9-10900X CPU is manufactured by Intel, while the RTX3080Ti graphics card is manufactured by NVIDIA. Intel is headquartered in Santa Clara, CA, USA, and NVIDIA also has its headquarters there. For training, the input image size was 416 × 416. A total of 200 epochs were used to train the network. The learning rate is 0.001 for the first 25 epochs, 0.0001 for epochs 25 to 100, and 0.00001 for epochs 100 to 200. A total of 1000 iterations are performed for each of the first 25 epochs. After 25 epochs, 2000 iterations are performed for each epoch. To achieve higher accuracy and generalization performance, data enhancement is performed on underwater data. By introducing color dithering, horizontal flipping, random rotation, and random cropping, it adapts to the color state changes of underwater targets under different shooting conditions.

### 4.2. Evaluation Metrics for Model Algorithms

The performance of the network is objectively evaluated using three metrics: frames per second (FPS), average precision (AP), and mean average precision (mAP) as follows:

(1) The speed of network detection is evaluated using the FPS metric, with higher values indicating faster network detection;
(2) The *AP* metric is used to evaluate the detection accuracy of each category, which can be calculated as follows:

(10) $$AP=\int_{0}^{1}P(R)dR$$

where *P* is precision, the probability that all detected targets are correctly detected, and *R* is recall, the probability that all true positives are correctly detected, which can be calculated as follows:

(11) $$P=\frac{TP}{TP+FP},\;R=\frac{TP}{TP+FN}$$

where *TP* is the number of positive samples correctly recognized, *FP* is the number of samples recognized as positive but actually negative, and *FN* is the number of samples recognized as negative but actually positive;

(3) The combined detection accuracy of the model is evaluated using *mAP*, and the calculation can be expressed as follows:

(12) $$mAP=\sum_{i=1}^{N}AP_{i}/N$$

where *N* is the number of categories and *AP_i_* is the average precision of category *i*;

(4) The model complexity uses parameters, and the specific calculation formula is as follows:
  (13) $$Params=C_{o}\;\times \;(k_{w}\;\times \;k_{h}\;\times \;C_{i}+1)$$
  where *C_o_* represents the number of output channels, *C_i_* represents the number of input channels, and *k_w_* and *k_h_* represent the width and height of the convolution kernel, respectively.

### 4.3. Experiments on Underwater Image Datasets

The underwater image datasets URPC2020 and URPC2018 contain 5543 and 4000 images, respectively, covering four classes of marine life, namely holothurian, starfish, echinus, and scallop. Due to the realism of the marine environment, these images typically suffer from low resolution, low signal-to-noise ratio, low contrast, color aberration, and blurred feature information, resulting in the loss of clear contour and texture information, limiting the accuracy of target identification and greatly reducing the effectiveness of our method. In addition, targets are dense and often obscured, presenting significant challenges to target detection. In underwater imagery, the scale of marine life is often small. Of particular note is the fact that some marine organisms, such as sea cucumbers and scallops, have protective colors that blend in with their environment and can hide. Also, due to the aggregation behavior of marine life, there is often a high density of targets in the captured images. All these characteristics make the task of underwater target detection difficult. In order to validate our proposed algorithm, we conducted a series of experiments on an underwater image dataset.

#### 4.3.1. Analyze the Effect of Image Resolution and Signal-to-Noise Ratio on Our Algorithm

In underwater environments, light propagation is affected by water absorption and scattering, resulting in distorted and blurred images. With low resolution, target details may not be clearly visible in the image, affecting the accuracy of target detection. Higher-resolution images can provide more detailed information that can help identify and locate underwater targets. In addition, the scattering and absorption of light introduces noise that can degrade image quality and obscure the target. If the signal-to-noise ratio is low, the target is drowned out by the noise, making it difficult for the detection algorithm to identify it correctly.

Looking at the experimental results in Table 2, the algorithm achieves an average detection accuracy of 80.55% when the input size is 384 × 384. When the input size is increased to 416 × 416, the average detection accuracy reaches 81.17%, which is a 0.62% improvement in detection accuracy. Experiments on the URPC2020 underwater image dataset show that a larger input size can provide finer target information, thus improving target detection accuracy.

Observing the experimental results in Table 3, we add Gaussian noise, which has a greater impact on the underwater image (“+” indicates the addition of Gaussian noise); by adding Gaussian noise, the clarity of the image decreases, reducing the quality and legibility of the image. Gaussian noise reduces the overall quality of the image, thus limiting the effective resolution of the image. Although the image has a certain number of pixels, some details and features cannot be clearly represented due to the presence of noise. Experiments on the URPC2020 underwater image dataset show that the proposed algorithm achieves a detection accuracy of 81.17%, whereas after adding noise to the image, the accuracy rate of the algorithm in this paper decreases by 1.39%.

**Figure 11:**
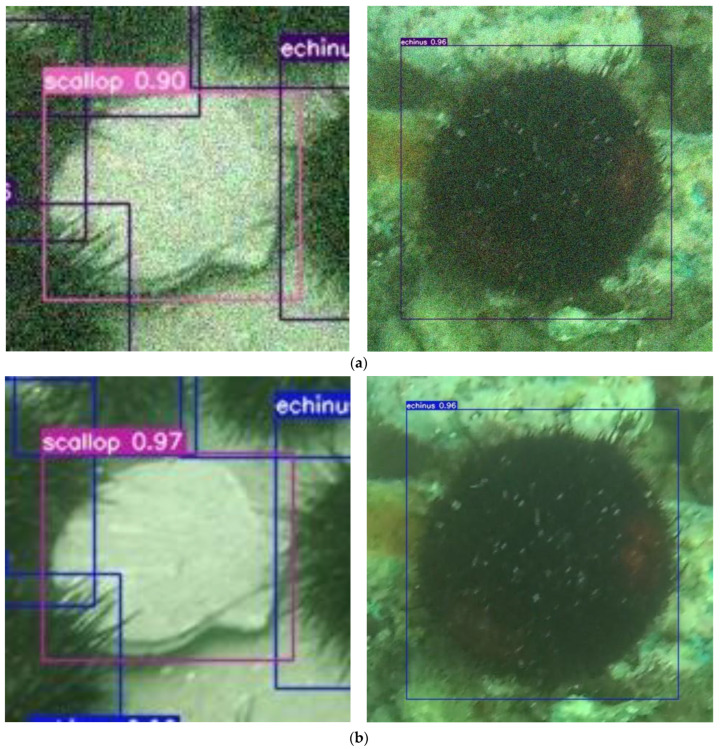
The comparison result of adding Gaussian noise and not adding Gaussian noise. (**a**) Detection results with Gaussian noise added. (**b**) Detection results without Gaussian noise added.

As can be seen in Figure 11, our algorithm can correctly detect and identify underwater organisms. The introduction of Gaussian noise in the underwater image reduces the effective resolution of the image, which affects the accuracy of the target detection algorithm. Due to the presence of noise, some details of the target features are missed, resulting in a decrease in target detection accuracy.

In summary, underwater target detection is affected by resolution and the signal-to-noise ratio, making detection difficult. Reducing the input resolution can lead to distortion or loss of some small targets, reducing the detection accuracy of small targets. Increasing the input resolution provides more target detail and helps to detect small targets. Higher-resolution images provide more accurate target localization, allowing the position of the target to be determined more accurately. Higher-resolution images reduce noise and interference in the image, reducing the likelihood of false detections and improving the reliability of the algorithm. In addition, a lower signal-to-noise ratio makes the noise in the image more prominent, blurring the features of the target in the image and, thus, reducing the visibility of the target. A low signal-to-noise ratio can lead to an increase in false alarms. Noise can be mistaken for a target, which can lead to false alarms in the algorithm, affecting the accuracy and reliability of detection. This has an impact on the ability to detect and locate targets in the underwater environment, which ultimately affects underwater target acquisition results.

#### 4.3.2. Comparison with Popular Object Detection Algorithms

We compared several popular target detection methods on the underwater image dataset URPC2020 and recorded the results as shown in Table 4 Our method is compared with Faster-RCNN, YOLOv5, YOLOv7, SSD, DSSD, SWIPENet, CornerNet, and CenterNet. Faster-RCNN is a two-stage target detection algorithm, while YOLOv5, SSD, and RetinaNet are classical one-stage target detection algorithms. SWIPNet is the most commonly used underwater target detection algorithm. We evaluate the effectiveness of target detection in terms of mean average precision (mAP) and frames per second (FPS). As can be seen in Table 4, Faster-RCNN achieves 71.45% mAP when implemented with VGGNet and 74.82% mAP when implemented with ResNet-101 + FPN, while the FPS decreases. However, YOLOv5 achieved 78.34% accuracy on mAP and had an FPS of 14.6, while YOLOv7 achieved 79.49% accuracy on mAP and an FPS of 17.5. The YOLOv5 and YOLOv7 methods showed high performance in terms of detection accuracy and speed. SSDs and DSSDs are almost equal in performance. SWIPTNet was used as a novel re-sampling weighted underwater algorithm to reduce the effect of underwater noise, but performance was average. The detection accuracy of the anchor-free detector CornerNet was significantly lower. This difference can be attributed to the fact that the above algorithms require several key corner points to be defined for underwater target detection and grouped according to the distance between these corner points. However, due to the presence of strong occlusion and a large number of small targets in the underwater environment, the problem of misgrouping can easily occur, which significantly reduces the detection accuracy. The method proposed in this paper achieves a detection accuracy of 81.17% with an FPS of 7.4, which meets the real-time requirement. In the following, we further analyze the advantages of the method.

In order to evaluate the effectiveness of the algorithm proposed in this paper, experiments were performed on the URPU2020 underwater object detection dataset, and we compared and analyzed its model complexity with that of the CenterNet algorithm. The experimental results are presented in Table 5. Using identical experimental settings, the proposed algorithm has a much lower model size, fewer model parameters, and fewer floating-point operations compared to the CenterNet algorithm. Specifically, the proposed algorithm’s model size is reduced by 638.5 M (i.e., compressed by 84.4%), the number of model parameters is reduced by 159.6 M (i.e., compressed by 84.3%), and the floating-point operation volume is reduced by 138.3 G (i.e., scaled down by 81.6%). These results demonstrate that the algorithm proposed in this paper significantly reduces the model complexity, making it more favorable for practical applications.

In addition, this paper performs experiments on the underwater image dataset URPC2018 to compare with the baseline network. The results are presented in Table 6. From Table 6, it can be seen that the algorithm in this paper outperforms the baseline network with faster detection speed, obtaining 77.02% mAP on URPC2018. This further demonstrates the effectiveness of the proposed parallel high-resolution network.

Table 7 displays detection results on the underwater image dataset URPC2018. SWIPTNet is a novel re-sampling weighted underwater algorithm used to reduce the effect of underwater noise; its detection accuracy was only 68.0%. The RoIMix [45] data enhancement is used to improve the detection of overlapping, occluded, and blurred targets; its detection accuracy reached 74.9%. It can be seen that the algorithm in this paper has a great advantage in detection accuracy, surpassing existing underwater target detection algorithms.

#### 4.3.3. Ablation Experiments

To validate the algorithms in this paper, we perform ablation experiments to compare and analyze the detection accuracy and speed. Table 8 shows the results of the ablation experiments on the URPC2020 underwater dataset. CenterNet was used as the baseline for the experiments on the underwater dataset.

The first row represents the baseline network with Hourglass-104 as the backbone network. The second row replaces the backbone network with HRNet, which slightly reduces the average accuracy but improves the detection speed, further demonstrating that HRNet is a lighter network that reduces model complexity and improves detection speed. With the addition of the enhanced attention module A-CBAM in the third row, excellent spatial information modeling capability is achieved by modeling the two-dimensional space in the activation function stage, capturing complex feature distributions and demonstrating feature enhancement in the spatial and channel dimensions, which improves the quality of the feature maps extracted by the network and increases the detection accuracy as well as the speed of inference. The fourth line introduces a receptive field augmentation module, which makes full use of the semantic and positional information of the network to further improve accuracy while maintaining detection speed.

In the evaluation of detection accuracy presented in Table 8, the categories corresponding to echinus and starfishes exhibit a more substantial number of targets and more distinctly delineated feature information. Therefore, under different experimental settings, the algorithm in this paper can achieve 91.32% and 85.32% accuracy for echinus and starfishes, respectively, because the model can easily extract target features and achieve high detection accuracy. However, the total number of targets in the two categories of holothurians and scallops was low. As these two target types are usually small in size and have ambiguous feature information, their detection accuracy is low. The detection accuracy of the algorithm in this paper for these two types of targets reached 76.40% and 71.67%, respectively, which is significantly higher than that of other networks. This indicates that the feature extraction capability of the network is significantly improved by the combination of different enhancement strategies proposed in this paper. This alleviates the limitations imposed by the lack of target data samples and improves the detection of small and fuzzy targets.

### 4.4. Experiments on PASCAL VOC Datasets

To further validate the performance of the algorithms in this paper, we conducted experiments on the PASCAL VOC dataset. We used the VOC2007 and VOC2012 training sets to train the network model and tested the network model on the VOC2007 test set. Table 9 shows the results of our method in comparison to currently popular target detection algorithms. The methods compared include one-stage and two-stage target detection algorithms. In general, two-stage target detection algorithms have higher detection accuracy but slower detection speed. For example, the Faster R-CNN using ResNet-101 improved the mAP from 73.2% to 76.4% compared to the VGGNet backbone. However, the single-stage target detection algorithm has the advantage of fast detection. For example, SSD512 has a detection speed of 19 FPS and YOLOv5 has a detection speed of 38 FPS. In addition, we can sacrifice some detection speed for higher detection accuracy. When the input size is 416 × 416, the mAP of the algorithm in this paper reaches 82.5%, which is higher and faster than the Faster R-CNN two-stage target detection algorithms; when the input size is increased to 512 × 512, the average detection accuracy reaches 82.9%, which is higher than that of the current mainstream single-stage target detection algorithms SSD, YOLOv5, and RetinaNet; the detection speed is reduced but the detection accuracy is higher. This shows that the algorithm in this paper still has advantages in the detection of general-purpose objects, which proves the reasonableness of the network structure design in this paper.

### 4.5. Visualization Analysis of Detection Results Output

**Figure 12:**
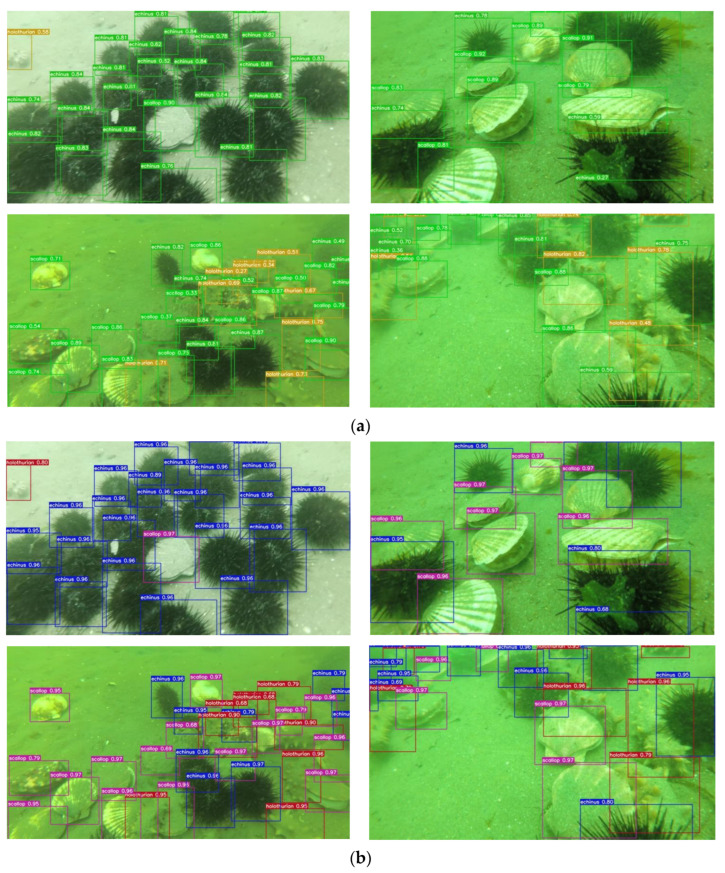
Comparison results between the algorithm in this paper and the baseline in the underwater image dataset URPC2020. (**a**) Baseline. (**b**) Ours.

In order to show the detection results of the algorithm in a more intuitive way, four images from the more complex dataset were randomly selected and compared with the original model, as shown in Figure 12. Echinus, scallops, starfishes, and holothurians are still relatively blurred underwater and highly integrated with the background, making detection difficult. The algorithm in this paper was able to correctly detect the target underwater marine organisms with high accuracy, proving the effectiveness of the improved model.

**Figure 13:**
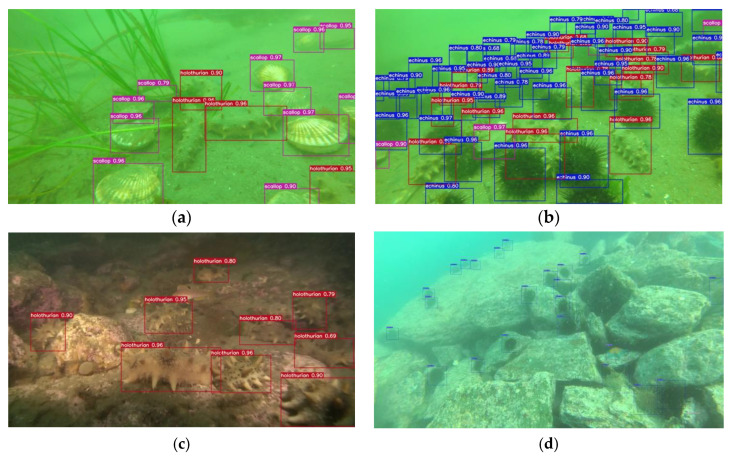
Detection results of different scenarios. (**a**) Seagrass disturbance. (**b**) Target overlap. (**c**) Complex background. (**d**) Long range small target.

As shown in Figure 13. By analyzing the above four images, we can conclude that the algorithm in this paper has good detection capability in the face of seagrass clutter, complex environments, and blurred overlapping targets. The comprehensive evaluation of the experimental results shows that the surface algorithm is a detection model with high generalization ability and can accurately detect targets of different states. It can be concluded that the algorithm in this paper has excellent comprehensive performance in underwater target detection. Based on the above results, further comparisons with the state of the art are made.

### 4.6. Comparison of the Results Obtained with the State of the Art

Based on the above results, further analysis and comparison with existing techniques are performed. The overall performance of our method is improved, and we compare the results of experiments on the underwater dataset URPC2020; is the performance of our method is higher than that of other mainstream target detection algorithms in terms of detection accuracy and higher than the latest YOLOv7 model’s detection accuracy. This shows that the improved algorithm in this paper has more advantages in detecting underwater targets and can achieve higher detection accuracy. It is worth noting that in the detection of underwater images, anchor-free frame detector CornerNet’s detection accuracy is significantly lower than that of this paper’s algorithm and other algorithms. To analyze the reasons why, the above algorithm needs to define a number of key corner points and group them according to the distance between the key points; underwater targets with severe occlusion are present; a wide range of small targets are present; and it is easy to misgroup, significantly reducing the detection accuracy from the point of view of detection speed. In terms of detection speed, the algorithm in this paper is faster than SWIPTNet, Faster R-CNN, CornerNet, and CenterNet, but slower than SSD, RetinaNet, YOLOv5, and YOLOv7 algorithms. The reason for this is that the backbone network of this paper is based on HRNet, which has fewer parameters and improves the detection speed, but due to the repeated multi-scale feature fusion, it is easier to misgroup underwater targets, which significantly reduces the detection accuracy. However, due to the repeated multi-scale feature fusion within the network, the network inference speed is reduced to some extent; in terms of model complexity, the model size, number of parameters, and floating-point operation volume in this paper are only 117.6 M, 29.7 M, and 31. 2 G, which are much lower than those of the benchmark network, indicating that the improved network in this paper is a relatively lightweight network, which can significantly reduce the number of parameters in the model, reduce the complexity of the model, and improve the network inference speed.

Replacing Hourglass-104 with HRNet as the backbone of the network results in a slight decrease in average accuracy and an increase in inference speed, further suggesting that HRNet is a lighter network that reduces model complexity and improves recognition speed. With the addition of the enhanced attention module A-CBAM, excellent spatial information modeling capability is achieved by modeling the two-dimensional space in the activation function stage, capturing complex feature distributions and demonstrating feature enhancement in the spatial and channel dimensions, which improves the quality of the feature maps extracted by the network and significantly improves both detection accuracy and inference speed. The receptive field augmentation module is introduced to fully exploit the semantic and positional information of the network to further improve the accuracy while maintaining detection speed.

For each type of detection accuracy, both echinus and starfishes have a larger number of targets and clearer feature information. Therefore, under different experimental settings, the model can easily extract target features and achieve high detection accuracy, and the algorithm in this paper can achieve 91.32% and 85.32% accuracy for echinus and starfishes, respectively. However, the total number of targets in the two categories of holothurians and scallops is small. As these two target types are usually small and have fuzzy feature information, their detection accuracies are lower. However, the algorithms in this paper achieve detection accuracies of 76.40% and 71.67% for these two types of targets, which is significantly higher than that of other networks. This indicates that the feature extraction capability of the network is significantly improved by the combination of different enhancement strategies proposed in this paper. To a certain extent, this alleviates the limitation of the scarcity of target data samples and improves the detection ability for small and fuzzy targets.

Comprehensively analyzed, the algorithm in this paper achieves the highest detection accuracy compared to other mainstream algorithms, while maintaining a faster detection speed and a lower number of model parameters, which has a significant advantage in detecting underwater targets.

## 5. Conclusions

To solve a number of problems such as complex underwater scenes and difficult target feature extraction, this paper makes targeted improvements to the CenterNet benchmark framework and proposes a parallel high-resolution underwater target detection algorithm. First, HRNet, a lighter high-resolution human posture estimation network, is used to improve the target feature representation and effectively reduce the semantic information lost in the image sampling process. Then, the attention module (A-CBAM) is enhanced to achieve pixel-level spatial information modeling capability by introducing the FReLU activation function, which captures complex feature distributions by modeling the two-dimensional space in the activation function stage. Feature enhancement in the spatial and channel dimensions is performed to improve understanding of fuzzy targets and small target objects and to better capture irregular and detailed object layouts. Finally, a receptive field augmentation module (RFAM) is constructed to obtain sufficient semantic information and rich detail information to further enhance the robustness and discrimination of features and improve the detection capability of the model for multi-scale underwater targets. The method improves the mAP by 4.96%, 2.62%, and 2.2% on the three publicly available datasets URPC2020, URPC2018, and PASCAL VOC2007, respectively. The experiments on the underwater target detection dataset URPU2020 show that the number of model parameters of the model in this paper is reduced by 159.6 M and compressed by 84.3% compared with the benchmark network. These results show that the algorithm proposed in this paper improves the accuracy and reduces the model complexity, which makes it more conducive to practical applications. Meanwhile, the surface algorithm is not only applicable to the underwater environment, but also to the surface environment. The strong generalization ability of the algorithm is verified.

There are several threats to the study of underwater object detection that affect the validity of the work in this paper. At the model level, the use of parallel high-resolution networks and feature extraction processes leads to problems of information loss and redundant parameters. The feature extraction capability of the high-resolution network is also somewhat lacking. A receptive field augmentation module was introduced but, at the same time, it increased the complexity of the model and reduced the speed of model inference. From an image point of view, the number of two types of targets, holothurians and scallops, is low. Since these two types of targets are usually small in size and have ambiguous feature information, their detection accuracy is low.

Future research will focus on improving model performance, increasing underwater image datasets, further exploring the potential of convolutional neural networks for marine target detection, and improving detection of fuzzy and small targets, as well as performance in turbid and crowded environments. Methodologically, we will investigate techniques such as model pruning and knowledge distillation and continue to explore lightweight processing approaches to improve the detection speed of the model.

## Figures and Tables

**Table 1:** Methods for summarizing related work.

Method	Year	Key Features
Zhou et al. [28]	2017	The combination of image enhancement techniques and an extended VGG16 feature extraction network is presented.
Villon et al. [29]	2020	Combined HOG and SVM.
Chen et al. [30]	2020	A novel sample distribution-based weighted loss function called IMA is introduced.
Ma et al. [31,32]	2019	Lightweight models were developed by pruning the SDD target detection model or integrating the MobileNet lightweight backbone.
Li et al. [33]	2022	Added an extra detection header to the YOLOv5 model.
Zhang et al. [34]	2022	Incorporated attentional mechanisms to learn about target features and ways to increase the receptive field.
Yao et al. [35,36]	2019	Used a residual network as a backbone.
Jiang et al. [37]	2021	A method called the Channel Sharpening Attention Module (CSAM) is proposed.

**Table 2:** Comparison results for different input sizes.

Network	Backbone	Input Size	AP (%)				mAP (%)
			Echinus	Holothurian	Scallop	Starfish	
Ours	AR-HRNet	416 × 416	91.32	76.40	71.67	85.32	81.17
Ours	AR-HRNet	384 × 384	91.89	75.34	70.86	84.13	80.55

**Table 3:** Comparison of detection accuracy with and without the addition of Gaussian noise.

Network	Backbone	AP (%)				mAP (%)
		Echinus	Holothurian	Scallop	Starfish	
Ours	AR-HRNet	91.32	76.40	71.67	85.32	81.17
Ours+	AR-HRNet	89.47	75.23	69.59	84.83	79.78

**Table 4:** Comparison results with popular object detector algorithms on URPC2020.

Network	Backbone	AP (%)				mAP (%)	FPS
		Echinus	Holothurian	Scallop	Starfish		
Faster R-CNN	ResNet-101 + FPN	86.33	63.17	69.25	80.56	74.82	3.4
Faster-RCNN	VGG-16	80.63	64.01	62.78	78.38	71.45	6.16
YOLOv5	CSP-Darknet53	87.71	67.69	75.33	81.63	78.34	14.6
YOLOv7	CSP-Darknet53	86.64	70.28	77.81	83.25	79.49	17.5
SSD512	VGG-16	84.50	69.30	66.90	80.50	75.30	14.3
DSSD	Residual-101	83.62	70.35	65.63	79.72	74.83	13.2
SWIPENet	VGG-16	84.32	70.75	49.62	79.23	70.98	9.8
RetinaNet	ResNet-50	83.42	62.83	69.03	80.94	74.05	9.56
CornerNet	Houglass-104	65.72	52.21	57.21	60.48	58.90	3.1
CenterNet	Houglass-104	86.43	69.78	68.07	80.59	76.21	5.3
Ours	AR-HRNet	91.32	76.40	71.67	85.32	81.17	7.4

**Table 5:** Comparison with CenterNet algorithm model complexity.

Network	Backbone	Size/M	Params/M	GFLOPs
CenterNet	Houglass-104	756.1	189.3	169.5
Ours	AR-HRNet	117.6	29.7	31.2

**Table 6:** Comparison results with popular object detection algorithms on URPC2018.

Network	Backbone	AP (%)				mAP (%)	FPS
		Echinus	Holothurian	Scallop	Starfish		
CornerNet	Houglass-104	61.45	45.96	51.21	53.37	50.90	3.1
CenterNet	Houglass-104	86.94	69.92	58.37	82.37	74.40	5.3
Ours	AR-HRNet	87.71	72.54	67.26	80.59	77.02	7.4

**Table 7:** Comparison with existing underwater object detection algorithms on URPC2018.

Network	Backbone	mAP (%)
SWIPTENet	SWIPTENet	68.0
ROIMix	ResNet-101	74.9
Ours	AR-HRNet	77.02

**Table 8:** Ablation experiments on URPC2020.

HRNet	A-CBAM	RFAB	Echinus	Holothurian	Scallop	Starfish	mAP (%)	FPS
			86.43	69.78	68.07	80.59	76.21	5.3
√			85.24	69.28	66.46	79.32	75.07	8.3
√	√		88.26	73.32	68.52	82.57	78.16	7.8
√	√	√	91.32	76.40	71.67	85.32	81.17	7.4

**Table 9:** Detection results on the PASCAL VOC2007 datasets.

Network	Backbone	Input Size	FPS	mAP (%)
Faster R-CNN	ResNet-101	1000 × 600	5	76.4
Faster R-CNN	VGG-16	1000 × 600	6.7	73.2
YOLOv5	CSP-Darknet53	416 × 416	38	78.9
SSD512	VGG-16	512 × 512	19	76.8
DSSD	ResNet-101	312 × 312	9.5	78.6
RetinaNet	ResNet-50	1000 × 600	7.6	74.8
CenterNet	ResNet-101	416 × 416	26	78.7
CenterNet	Houglass-104	416 × 416	5	80.7
Ours	AR-HRNet	416 × 416	8	82.5
Ours	AR-HRNet	512 × 512	7.8	82.9

## Data Availability

Not applicable.
